# Simvastatin improves the sexual health-related quality of life in men aged 40 years and over with erectile dysfunction: additional data from the erectile dysfunction and statin trial

**DOI:** 10.1186/1471-2490-14-24

**Published:** 2014-03-05

**Authors:** Daksha Trivedi, David M Wellsted, Jade B Collard, Michael Kirby

**Affiliations:** 1 Centre for Research in Primary and Community Care, University of Hertfordshire, Hatfield, UK; 2 Centre for Lifespan and Chronic Illness Research, University of Hertfordshire, Hatfield, UK

**Keywords:** Erectile dysfunction, Statins, Sexual health quality of life, Randomised controlled trial

## Abstract

**Background:**

Erectile dysfunction is prevalent in men over 40 years, affecting their quality of life and that of their partners. The aims of this study were:

a) To evaluate the internal reliability of the male erectile dysfunction specific quality of life (MED-QoL) scale and explore its factor structure.

b) To evaluate the effect of simvastatin on subscales of the MED-QoL in men over forty years with erectile dysfunction.

**Methods:**

This is a double blind randomised controlled trial of 40 mg simvastatin or placebo given once daily for six months to men over forty years with untreated erectile dysfunction, who were not at high cardiovascular risk and were not on anti-hypertensive or lipid-lowering medication. 173 eligible men were recruited from 10 general practices in East of England. Data were collected at two points over 30 weeks.

We report on the factor structure of MED-QoL, the internal reliability of the scale and the derived subscales, and the effect of simvastatin on MED-QoL subscales.

**Results:**

An initial analysis of the MED-QoL items suggested that a number of items should be removed (MED-QoL-R). Exploratory factor analysis identified three subscales within the MED-QoL-R which accounted for 96% of the variance, related to feelings of Control, initiating Intimacy, and Emotional response to erectile dysfunction. The alpha value for the revised scale (MED-Qol-R) was >0.95 and exceeded .82 for each subscale. Regression analysis showed that patients in the placebo group experienced a significantly reduced feeling of Control over erectile dysfunction than those in the statin group. Those in the placebo group had significantly lower Emotional response than those in the statin group at the close of trial, but there was no significant treatment effect on Intimacy.

**Conclusions:**

Our revised MED-QoL-R identified three subscales. Secondary analysis showed a significant improvement in sexual health related quality of life, specifically in relation to perception of control and emotional health in men with untreated erectile dysfunction given 40 mg simvastatin for six months.

**Trial registration:**

Current Controlled Trials ISRCTN66772971.

## Background

Erectile dysfunction (ED) is the consistent inability to achieve or maintain an erection that is sufficient for satisfactory sexual intercourse. Although ED affects sexual and mental health [1,2], the rates of consultation for ED remain low [3,4] and not all patients respond to Sildenafil and other phosphodiesterase inhibitors [5]. We reported the results of a randomised controlled trial (RCT) evaluating the effectiveness and cost effectiveness of simvastatin therapy in men with ED in men with untreated ED, but without significant cardiovascular risk factors [6]. The lipid lowering drug simvastatin was chosen for this study because ED shares risk factors with cardiovascular disease (CVD) [7-11]. It is associated with high total and low density lipoprotein cholesterol [12-14] and endothelial dysfunction.

There is a consensus that ED is either a predictor of future CVD or an early marker of silent atherosclerotic cardiovascular disease [9,10]. Small scale studies have indicated that atorvastatin can reduce ED and improve sexual function [12,15] but to date, there is no evidence to suggest that statins improve sexual health related quality of life in men with untreated ED.

In our previously published trial [6], there was a nonsignificant change in erectile function due to simvastatin treatment, although patients with more severe ED at baseline showed a larger improvement than patients with mild/moderate ED. However, simvastatin significantly improved the male ED-specific quality of life (MED-QoL), LDL cholesterol and reduced future cardiovascular risk [6]. It remains unclear why the MED-QoL improved significantly with only a small effect on erectile function, as the sexual encounter profile data showed nonsignificant treatment effect on satisfaction or success.

The MED-QoL scale has not been widely used, and there are few publications addressing the measurement of sexual health related quality of life at all using the MED-QoL. Only one paper has published any evidence of the scale reliability or validity using a sample of 69 men [16]. The current analysis aims to evaluate the internal reliability of the MED-QoL scale, its factor structure, and the extent any identified factors show drug related changes.

## Methods

This is a secondary analysis of data from a double blind RCT comparing treatment with simvastatin or placebo on ED conducted in ten general practices in the East of England. The study design methods and analysis for the main study have been published previously [6,17]. The study protocol was approved by the Essex 1 Research Ethics Committee and clinical trial authorisation was obtained from the UK Medicines and Healthcare Products Regulatory Agency.

### Main trial study design

#### Patients

173 eligible men aged forty years and over with untreated ED (score < 22 on the IIEF-5 (International Index of Erectile Function)), and no other cardiovascular risk factors were randomised to receive 40 mg simvastatin or placebo once daily for six months. Exclusions were based on participants’ medical and drug history and those considered by the GP to be unsuitable, such as significant CVD risk requiring statin treatment.

#### Study design

Randomization to active simvastatin or placebo was on a 1:1 basis and was computer-generated at the Clinical Trials Co-ordinating Centre. Data were collected at baseline, 3 months and 6 months at assessment visits to the GP clinic. At each visit, participants completed the IIEF-5 questionnaire, the MED-QoL and the EQ-5D [16,18,19]. Sexual encounter profile (SEP) diaries were completed whenever an encounter occurred and were collected at each assessment. All questionnaires were completed by the men at the assessment visits in a private area, and were given support if necessary to clarify any raised issues.

The MED-QoL questionnaire consists of 27 items. All domains assess the effects of erectile dysfunction on the respondents’ quality of life. The scale is based on 4 response categories: ‘Not at all’, ‘A little’, ‘Quite a lot’ and ‘Very much’. All 27 items in the MED-QoL are listed in Table 1.

**Table 1:** Exploratory factor analysis of the MED-QoL

**Factor**	Item*	**Factor 1**	**Factor 2**	**Factor 3**	**Cumulative variance**	**Alpha**	**Eigenvalue**
1 (Control)	1. I feel sexually frustrated because of my erection difficulties	0.638*	0.100	0.302	0.37	0.82	11.40
	2. My problem makes me feel depressed	0.515*	0.119	0.443			
	3. I feel less of a man because of my problem	0.576*	0.296	0.448			
	4. I’ve lost confidence in my sexual ability	0.676*	0.349	0.211			
	5. I worry that I won’t be able to get or keep up an erection	0.644*	0.294	0.302			
	7. I feel that I have lost control over my erections	0.681*	0.230	0.163			
	10. I worry about the future of my sex life	0.512*	0.417	0.327			
	12. I’m embarrassed about my problem	0.532*	0.236	0.480			
	16. I get less pleasure from life because of my erection problem	0.502*	0.401	0.230			
2 (Intimacy)	11. I’ve lost pleasure in sex	0.454	0.569*	0.070	0.69	0.85	1.54
	17. I feel guilty about my erection problem	0.219	0.521*	0.478			
	18. I am afraid to “make the first move” with my partner	0.316	0.665*	0.127			
	19. I worry that my partner blames herself for my problem	0.062	0.540*	0.305			
	20. I worry that I’m letting my partner down	0.439	0.547*	0.427			
	21. I worry that I am not satisfying my partner sexually	0.506	0.521*	0.217			
	22. I worry that we are going apart because of my problem	0.257	0.605*	0.265			
	25. I worry that she thinks I don’t want her because of my problem	0.194	0.690*	0.300			
3 (Emotion)	6. My erection problem is always on my mind	0.459	0.302	0.506*	0.96	0.86	1.02
	8. I blame myself for my problem	0.205	0.381	0.566*			
	9. I feel angry because of my problem	0.313	0.168	0.676*			
	13. I worry about being humiliated because of my problem	0.263	0.288	0.626*			
Excluded	14. I try to avoid having sex	NA	NA	NA			
	15. I feel different from other men because of my erection problem	NA	NA	NA			
	23. I worry that she is looking for someone else because of my problem	NA	NA	NA			
	24. I feel that she blames me for my erection problem	NA	NA	NA			
	26. I have trouble talking to her about my problem	NA	NA	NA			
	27. My erection problem interferes with my daily activities	NA	NA	NA			
Scale total					.96	.95	

MED-QoL-R: male erectile dysfunction-specific quality of life revised scale. *Item numbers are based on the original MED-QoL scale. Factor analysis with varimax rotation, excluding items 14, 15, 23, 24,26 and 27.

### Analysis

The primary analysis of the data from this trial has been reported previously [6] using the original MED-QoL questionnaire [16].

### Secondary data analysis: MED-QoL scale reliability

A step wise approach was taken to evaluate the reliability and factor structure of the MEDQoL scale. In the first step (item reduction) items were evaluated for low mean and a lack of endorsement, and low item-total correlation. Identified items were examined to evaluate their face validity before removal. In the second step an exploratory factor analysis was undertaken, with Varimax rotation, and assessment of Kaiser-Meyer-Olkin (KMO) sampling adequacy. Several factors (subscales) were identified and the internal reliability of the items for each factor was assessed. Items were again considered for removal if the item-test statistics were weak, the factor loading was < .5 and if the KMO value indicated poor model fit.

This process generated a reduced scale, removing 6 items from the original MED-QoL scale. To avoid confusion we named the revised version the MED-QoL-R. The scores for each item in a subscale were summed and the subscale score normalised (0-100). The identified subscales were evaluated to determine the extent to which the drug related changes in the total MED-QoL-R scores were determined by particular subscales. Based on CONSORT guidelines, the difference in the subscale scores at close of trial was evaluated for each subscale in turn. A regression model was used in which the outcome was the MED-QoL-R sub-scale score with treatment arm (statin vs. placebo) as a predictor. The outcome was adjusted for baseline ED severity (and the interaction between severity and arm), baseline testosterone, baseline 10 year cardiovascular risk, and the baseline score for the subscale score. As some of the distributions were positively skewed, log transformation was applied to the data and bootstrapped confidence intervals were estimated (with 3000 replications).

## Results

In our trial, 173 patients were randomised, of which 131 patients completed some, and 113 completed all of the study outcome measures [6]. 60 (34%) patients withdrew from randomised treatment, but 18 of these patients continued to complete the QoL measures. The proportion withdrawing did not depend on study arm (χ^2^ (1) =0.5, p > 0.05).

Item numbers 15, 23, 24 and 27 of the 27 items had low mean response values (1.60, 1.21, 1.48 & 1.15, respectively), and had few responses in the highest category (<3% 'Very much'), indicative of a lack of endorsement of these items. On this basis these items were removed.

### Factor analysis

Following item reduction, the aim was to evaluate the underlying factor structure. An initial factor analysis was computed, excluding items 15, 23. 24 and 27 in order to assess the structure within the scale. Alongside the factor analysis, the Kaiser-Meir-Olkin (KMO) was also computed, assessing sampling adequacy of the overall scale and each item for factor analysis. A low KMO indicates that the item may not be relevant for the model specified and therefore may be a basis to exclude the item. For this model 3 factors with an Eigen value above 1 were found. Following the analysis, it was evident that as well as having a low mean, item 14 and 26 had a low KMO (0.88, 0.83, respectively). As item 14 already had a relatively low mean, it was excluded from analysis alongside items 15, 23,24 and 27 and the factor model was re-evaluated (Table 1).

### Reliability

Following factor analysis excluding items 14, 15, 23, 24 and 27, the internal reliability of each subscale was determined using Cronbach’s Alpha. Item 26 had low item-test reliability (0.58) compared to the rest of subscale 2 and was therefore removed from analysis. Subsequently, factor analysis with varimax rotation was computed once again, excluding items 14, 15, 23, 24, 26 and 27. The analysis yielded 3-factor model accounting for 96% of the variance (Table 1).

The factor loadings for each item followed a pattern, which we interpreted in the following way: Subscale (factor) 1 tended to belong to items which were related to feelings of control (Control); the items in subscale 2 were concerned with difficulties in initiating intimate contact (Intimacy); and the subscale 3 items were related to emotional response to erectile dysfunction (Emotion). Although there is a lot of overlap in the possible meaning of subscales 1 and 3, the 4 highest loading items on factor 1 are related to feelings of control ([item 1] frustration, [item 4] confidence, [item 5] worry, and [item 7] loss of control with the highest loading) rather than emotion, and we have therefor interpreted the factor in that way.

### Regression modelling: effectiveness on MED-QoL-R subscales

Our initial analysis showed that the improvement in IIEF due to statin treatment was not statistically different to placebo, although patients with severe ED at baseline showed a larger change than patients with mild/moderate ED [6]. There was a statistically larger improvement in total MED-QoL score for men on statin compared to placebo (5% change in total scale score vs. 2%, z = 2.09, p = .04), which was higher for men with severe ED (12% change in total scale score vs. 5%, z = 4.52, p < 0.001). In this analysis, using the MED-QoL-R, fitting a regression model on each factor (Control, Intimacy and Emotion) allows for an evaluation of changes in quality of life in relation to the three subscales highlighted in the factor analysis. Due to the distribution of the data, log transformations were applied to the outcome variables and the exponentiated values were obtained to better explain the linear relationship between variables (Table 2).

**Table 2:** Regression models evaluating the effect of statin treatment on men with ED (statin vs placebo groups) for each subscale (1-3) of the MED-QoL-R

	**N**	**Proportionate difference between study arms and 95% CI (regression model)**		**Mean values at close of trial (unadjusted values)**	
				**Severe ED**	**Mild/moderate ED**
**Total MED-QoL-R**	109	0.16 (0.01, 0.30)	**Statin**	66.1 (58.1, 74.1)	70.7 (62.4, 78.9)
			**Placebo**	58.3 (48.5, 68.2)	68.7 (61.6, 75.9)
**Feeling of control (Subscale 1)**	61^#^	0.40 (0.24, 0.53)*	**Statin**	60.7 (52.3, 69.2)	67.2 (59.6, 74.9)
			**Placebo**	51.6 (41.4, 61.9)	63.8 (56.6, 71.1)
**Initiating intimacy (Subscale 2)**	107	0.08 (0.12, 0.26)	**Statin**	67.3 (58.1, 76.6)	72.8 (63.8, 81.8)
			**Placebo**	62.7 (51.4, 74.1)	71.5 (64.0, 79.0)
**Emotion response (Subscale 3)**	104	0.25 (0.05, 0.41)*	**Statin**	75.7 (65.8, 85.5)	74.2 (64.2, 84.1)
			**Placebo**	64.4 (50.7, 78.0)	74.3 (65.9, 82.6)

MED-QoL-R: Revised male erectile dysfunction-specific quality of life. Column 3 provides the estimated proportionate difference between the study arms (1-exp(model coefficient). Thus 0.4 represents a 40% lower value in the Placebo arm compared to the Statin arm. Regression models (bootstrapped n = 3000) for each subscale were specified with treatment arm, ED severity, arm by severity, and baseline testosterone, cardiovascular risk, and time on treatment as variables. ^#^The number of patients who completed sufficient SEP Diaries (eg encounters) for inclusion in the analysis was lower than for most other outcomes-however for factor 1 (but not the other factors) the number of reported encounters was a very strong predictor of the outcome, and was therefore included in the model. *p < 0.05.

The regression model showed that for the Control subscale, the level of control over their ED perceived by those in the placebo group is significantly less than those in the statin treatment group at the close of trial (z = -3.15, p < 0.005). Similarly, those in the severe ED group have a significantly lower perception of control than those with mild/moderate ED (z = - 1.97 p = 0.05).

Uniquely for this subscale, an increased number of reported encounters between the couples at mid trial was related to the observed advantage for perceived control in men in the statin arm compared to the control arm. Inclusion of the number of encounters did compromise the number of patients entered into the model (n = 61) but was a strong predictor of the outcome that a decision was made to include the number of encounters in the model. The men excluded from the analysis (n = 47) did not substantially differ from the men included in the analysis (n = 61) on any of the major factors measured (e.g. age, ED severity, treatment arm). For the other two factors (Intimacy and Emotion) including the change in the number of reported encounters from baseline to close made no contribution to the model and was therefore not included.

The regression model fitted to the Intimacy subscale showed that there was no significant effect of treatment arm (z = -0.83 p > 0.05), ED severity (z = 0.66 p > 0.05), baseline testosterone levels (z = -0.05 p > 0.05), baseline cardiovascular risk (z = 1.31 p > 0.05) or an interaction between treatment arm and ED severity (z = 0.14 p > 0.05) on the level of intimacy between the participant and their partners.

The regression model for the Emotion subscale showed that those in the placebo group have significantly lower emotional state than those in the statin group (z = -2.35 p = 0.02) at study close. In general, the mild/moderate patients reported a higher emotional state than those with severe ED (z = -2.47 p = 0.01). There is a significant interaction between treatment arm and severity (z = 2.45 p = 0.01) indicating that patients in the severe ED group benefited significantly more from treatment than those in the mild to moderate ED groups.

## Discussion

Our initial analysis showed that simvastatin significantly improved the overall sexual health related quality of life (MED-QoL), which was largest for men with severe ED, despite the fact that the effect on erectile function was not significant [6]. We identified three subscales within MED-QOL that accounted for 96% of the variance relating to three quality of life characteristics: feelings of Control, problems initiating Intimacy and Emotion response to ED; named MED-QoL-R. Regression analysis showed that for the Control subscale, those with severe ED had significantly lower perception of control than those with mild to moderate ED. Similarly, those treated with statin had significantly improved perceptions of control at the close of trial than those treated with placebo. Similar results were found for subscale 3 (Emotion–excluding 4 outliers) where those treated with statin had a significantly more positive emotional state at the close of trial than those treated with placebo, although this is demonstrated mostly within the severe ED group. Those with mild to moderate ED had more positive emotional states than those with severe ED. The effect of statins on mood is equivocal [20] and it is possible that emotional improvements are mediated via improvement in ED.

Subscale 2, Intimacy, demonstrated no significant effect of either treatment arm or ED severity on level of intimacy and neither this nor Emotion were influenced by the number of encounters at midtrial. Uniquely, an increased number of reported encounters is a significant contributing factor in the improved perception of control over ED for men treated with simvastatin. This finding supports previous studies which show that more frequent erections improve erectile function, suggesting that men with frequent erections are likely to have better perception of control [21].

Our initial results showed no drug specific effect for SEP erections or satisfaction, although there was an increase in reported satisfaction over time for all participants and patients with mild/moderate ED reported greater satisfaction than those with severe ED. However, simvastatin showed no significant effect on SEP erections or satisfaction or reported frequency of encounters or IIEF severity, although participants attempting intercourse more than once a week on average reported higher IIEF scores at close of trial [6]. Our overall findings suggest that small changes in erectile function can have a significant impact on the sexual health related quality of life. This could be a result of an improvement in both the perception of control and emotional domains, but not a change in perceptions of intimacy.

The MED-QoL scale has previously not been extensively used, or tested for reliability and validity. To our knowledge the number of reported cases is less than 200 in total.

Undertaking an analysis of the reliability and factor structure of the scale, and evaluating drug related changes in the determined subscales from the same data limits the external reliability of the reported analysis. Care needs to be taken in interpreting these results until additional data samples can be collected and evaluated from a different population of men, demonstrating the external reliability of our findings.

Our objective in undertaking this analysis was to further evaluate the reliability and explore the factor structure of the MED-QoL scale, given that this had not been done before, and not reported in an external patient sample. Specifically we did not set out to create a brief measure. The scale created, the MED-QoL-R, has 21 items, and represents the most robust (e.g. reliable) scale suggested by the analysis of our data.

A number of scales to evaluate ED in men have been developed over the past 20 years [22-26]. The choice of the MED-QoL in our trial was based on familiarity of the scale amongst the research team. The current study offers additional evaluation of the MED-QoL in an external patient sample with recognised statistical methods, unlike most other scales developed. By undertaking this study in a sample drawn from a clinical trial of men not otherwise being treated, the analysis provides addition information about the reliability and underlying psychological dimensions of the MED-QoL, and provides a revised version of the scale, the MED-QoL-R.

We may have seen larger changes and more benefit if we had larger study sample, a longer treatment interval and a more potent statin. The total MED-QoL-R scores at close of trial showed that health related quality of life significantly improves when treated with statin than with placebo. Also, as found within the individual subscales, those with mild to moderate ED have significantly higher quality of life than those with severe ED. Overall the results demonstrate that sexual health related quality of life in males with ED is significantly influenced by drug treatment (statin compared to placebo) and by severity of the ED. Specifically, both feelings of control and emotional response to ED highlight this, compared to intimacy which is unaffected by treatment and severity of ED. The study excluded patients with significantly high CVD risk factors for ethical reasons. These were the men most likely to benefit from the intervention. Erectile function measured using the IIEF score defines ED principally in physical terms (hardness, penetration success), but does not take account of wider issues related to intimate relationships, which may or may not be related to cardiovascular health. It is possible that improving cardiovascular health has more general effects on sexual health which leads to improved sexual interactions that are not captured by the IIEF, but are captured by the MED- QoL-R, as seen in this additional analysis of subscales. There are also relationship issues to consider as enrolment in this study and contact with research nurses may have altered the sexual behaviour of these couples, and control as well as emotional health may be important. Research nurses involved in this study reported that relationships can be improved by the involvement of partners in the study. Further research into partner issues relating to sexual dysfunction is required. Routine consultations could include questions on ED as it has a big impact on quality of life of sufferer and partner. There is evidence to suggest that doctors need to support patients’ sexual activity as regular intercourse may improve erectile function and quality of life [21]. This is supported by our previous findings of a small positive correlation between frequency of intercourse and IIEF score [6].

## Conclusions

We have evaluated the MED-QoL and produced the revised MED-QoL-R with three identified subscales. Our secondary analysis has shown a significant improvement in sexual health related quality of life, specifically in relation to perception of control and emotional health in men with untreated ED given 40 mg simvastatin for six months. In addition our findings further demonstrate that the number of reported encounters may have a positive effect on perception of control over ED for men treated with simvastatin. Therefore, women could also be informed not only about the risks associated with ED and later development of CVD but also about their partners’ sexual health related quality of life and to encourage them to attend for medical assessment.

## Abbreviations

CVD: Cardiovascular disease; ED: Erectile dysfunction; IIEF-5: International index of erectile function; KMO: Kaiser-Meyer-Olkin; MED-QoL: Male erectile dysfunction specific quality of life; MED-QoL-R: Revised male erectile dysfunction specific quality of life; RCT: Randomised controlled trial; SEP: Sexual encounter profile.

## Competing interests

Michael Kirby has received funding for research, advice, lecturing, and conference attendance from Pharmaceutical Industry.

## Authors’ contributions

MK had the initial idea; DT coordinated the trial and has written the final manuscript. DW and JC have conducted the analysis. All authors have given final approval of this version for publication.

## Pre-publication history

The pre-publication history for this paper can be accessed here:

http://www.biomedcentral.com/1471-2490/14/24/prepub
